# Downregulation of oncogenic *RAS* and *c-Myc* expression in MOLT-4 leukaemia cells by a salicylaldehyde semicarbazone copper(II) complex

**DOI:** 10.1038/srep36868

**Published:** 2016-11-14

**Authors:** Yan-Yih Goh, Yaw-Kai Yan, Nguan Soon Tan, Su-Ann Goh, Shang Li, You-Chuan Teoh, Peter P. F. Lee

**Affiliations:** 1 Natural Sciences & Science Education, National Institute of Education, Nanyang Technological University, 1 Nanyang Walk, Singapore 637616, Singapore; 2 School of Biological Sciences, Nanyang Technological University, 60 Nanyang Drive, Singapore 637551; 3 Lee Kong Chian School of Medicine, Nanyang Technological University, 50 Nanyang Avenue, Singapore 639798, Singapore; 4 Institute of Molecular and Cell Biology, 61 Biopolis Drive, Singapore 138673, Singapore; 5 KK Women’s and Children’s Hospital, 100 Bukit Timah Road, Singapore 229899, Singapore; 6 Duke-NUS Medical School, 8 College Road, Singapore 169857, Singapore; 7 Singapore Institute of Technology, 10 Dover Drive, Singapore 138683, Singapore

## Abstract

Copper complexes with potent anti-tumor effect have been extensively developed. Most investigations of their modes of action focused on the biomolecular targets but not the signal transduction between target binding and cell death. We have previously shown that the cytotoxic complex pyridine(2,4-dihydroxybenzaldehyde dibenzyl semicarbazone)copper(II) (complex **1**) shows selective binding to human telomeric G-quadruplex DNA over double-stranded DNA *in vitro*. Herein, we elucidate the mechanism of action by which complex **1** induces apoptosis in MOLT-4 cells. Complex **1** accumulates in the nuclei and differentially downregulates the expression of *c-Myc, c-Kit* and *KRAS* oncogenes. Chemical affinity capture assay results show that the complex is associated with c-Myc and KRAS quadruplex sequences in MOLT-4 cells. We further showed that the reduction in Ras protein expression resulted in attenuated MEK-ERK and PI3K-Akt signalling activities, leading to the activation of caspase-dependent apoptosis. Notably, complex **1** increased the sensitivity of MOLT-4 cells to cisplatin and *vice versa*. Overall, we demonstrated that complex **1** induces apoptosis, at least in part, by suppressing *KRAS, c-Kit* and *c-Myc* oncogene expression and the pro-survival MEK-ERK and PI3K-Akt signalling pathways.

The initial success of cisplatin in the clinical treatment of a variety of cancers has placed coordination chemistry in the limelight in the fight against cancers. Even though cisplatin is highly effective in treating various cancers, its efficacy is limited by its side effects and intrinsic or acquired resistance^1^,^2^. This stimulated extensive research to develop various families of small molecules, based on different metals, and different targets, with improved pharmacological properties^3^,^4^. With the assumption that endogenous metals may be less toxic toward normal cells than cancer cells, copper-based anticancer complexes have been extensively investigated^5^,^6^. Strategies involving proteasome inhibition as well as DNA targeting in cancer therapies have been extensively studied^7^,^8^.

To date, most investigations focused on the ability of copper complexes to interact with duplex DNA, either through covalent bonding or non-covalent interaction^5^,^9^. In many cases, this interaction resulted in DNA oxidative cleavage through a Fenton-type reaction to generate high levels of reactive oxygen species (ROS)^10^. The cellular response to the DNA damage is the activation of diverse repairing mechanisms, the failure of which would trigger cell death. Despite numerous copper complexes being reported to trigger cell death due to DNA damage, little is known about the signal transduction mechanisms between complexes binding to DNA and apoptosis induction in cancer cells^5^,^6^. We have previously reported a series of square planar salicylaldehyde semicarbazone copper(II) complexes that showed high toxicity to cancer cells and acted via intercalating with DNA and generation of ROS^11^,^12^. Further derivatising of one of these complexes led to complex **1** (Fig. 1A), which binds selectively to telomeric G-quadruplex over double-stranded DNA^13^.

In this study, we elucidated the mechanism of action by which complex **1** induces apoptosis in MOLT-4 cells. We examined the subcellular distribution of complex **1** in MOLT-4 cells and determined its inhibitory effect on telomere extension using the telomeric repeat amplification protocol, measurement on telomeric lengths and locating induced double-strand breaks in the genomic DNA. The binding affinity of complex **1** to G-quadruplex containing promoter sequences of some oncogenes (*c-Myc*, *c-Kit, KRAS* and VEGF) and cancer-related genes (*TERT* and *HIF1α*) was then studied *in vitro*. Chemical affinity capture assay was also performed to investigate the association of complex **1** to telomere, *c-Myc* and *KRAS* quadruplex sequences *in situ*. The results correlate with the differential gene expression of these oncogenes and reduced protein levels observed in treated cells. In addition, the effect of reduced Ras protein level on downstream cellular proliferation MEK-ERK and anti-apoptotic PI3K-Akt signalling pathways was probed. Finally, the synergistic effect of complex **1** and cisplatin on the inhibition of MOLT-4 cells was investigated.

## Results

### Cellular translocation of complex 1 into the nucleus

**Figure 1:**
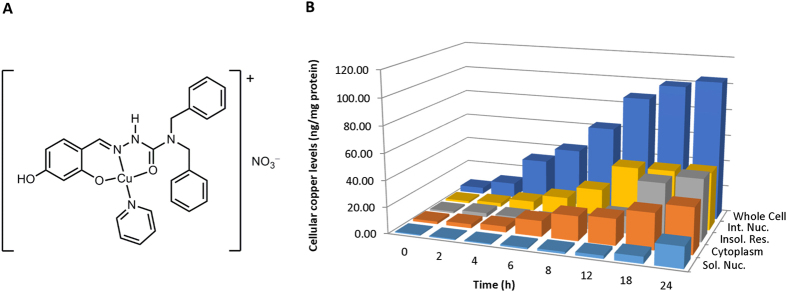
(**A**) Structure of complex **1**. (**B**) Cellular uptake data for complex **1**. The cellular copper levels are shown for whole cells, intact nuclei (Int. Nuc.), cytoplasm, soluble fraction of nuclei (Sol. Nuc.), and insoluble residue (Insol. Res.) remaining after extracting the cytoplasm and soluble nuclear fractions. Quantification data are represented as mean (n = 4).

To identify the major site of action of complex **1**, cellular uptake studies were carried out. In particular, we were interested to establish whether complex **1** enters the nucleus because it was designed to bind to G-quadruplexes. Thus, MOLT-4 cells were incubated over 24 h at 37 °C at the sub-lethal concentration of 3.0 μM. The copper content of the treated cells was determined for whole cell, intact nucleus, soluble cytoplasm and nuclear fractions, and insoluble residue, using graphite furnace atomic absorption spectrometry (GFAA). Cellular copper levels are expressed as ng of copper/mg of protein of cell and the results reported are a mean of four separate experiments for each data point (Fig. 1B). The copper complex was found to be taken up by MOLT-4 cells as early as 2 h after treatment, its level slowly increased between the 2^nd^ and 8^th^, surged at 12 h, and then approached a plateau at 18 h after treatment. Similarly, we found progressive increase in the amount of copper in the cytoplasm and the intact nucleus over the 24 h treatment. It is noteworthy that when we probed for the soluble portion of the nuclear fraction, the copper content remained low in first 8 h, increased slightly at 12 h, and only started to increase appreciably between 18 and 24 h. Most importantly, very little nuclear copper was located in the soluble nuclear fraction (e.g. 41.8 vs 2.4 ng copper/mg of protein found in intact vs soluble nuclear fraction at 12 h after treatment) suggesting that the copper complex was mostly bound to chromosomal DNA, rather than the soluble nucleoplasm fraction. To ensure cytotoxicity is not due to copper poisoning, we used 5.0 μM of CuSO_4_ treatment for the same duration. Results showed much higher intake of copper ion (308 ng copper/mg of protein) without any adverse effect (MTT IC_50 (24 h)_ > 125 μM) on MOLT-4 cells. The cellular uptake values are higher than those obtained for osmium(II)^14^ and platinum(II) complexes^15^ (2–3 ng /mg of protein after 24 h treatment in A2780 ovarian carcinomas and U2OS osteosarcomas respectively) previously reported.

### Complex 1 targets both telomeric and non-telomeric DNA

**Figure 2:**
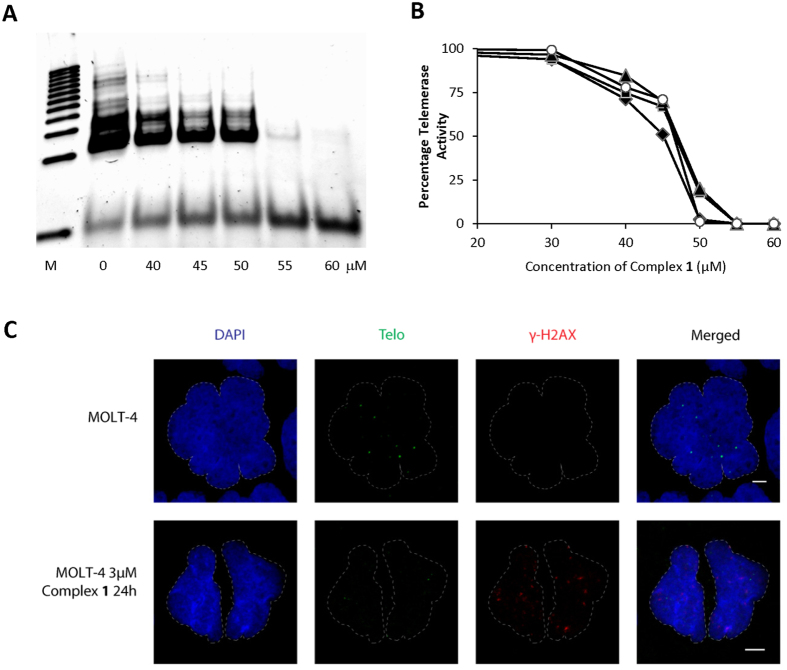
Telomeric extension inhibition by copper complex 1 in MOLT-4 cells extract. (**A**) Electropherogram of telomeric extension inhibition showing ladders generated by the action of telomerase on a TS primer after 33 PCR cycles using conventional TRAP assay with increasing concentrations of complex **1**. The lowest band in each lane was TS primer not incorporated for telomere extension. A representative image of four independent experiments is shown. (**B**) Telomerase activity in MOLT-4 cells measured using real time quantitative PCR (RTQ-TRAP) method. The activity was expressed as the percentage of the activity of the 0 μM solvent control treatment. The telomerase activity was calculated based on Ct values, taking the Ct value as 100% for 0 μM control and 0% when the fluorescence intensity did not reach the fluorescence threshold. Results from four independent experiments are plotted. (**C**) Immunofluorescence staining analysis of TelC and γ-H2AX foci in MOLT-4 cells (control) and cells treated with 3 μM of complex **1** for 24 h. The dotted white lines indicate the nuclear peripheries. The cell nuclei were stained with DAPI. Scale bar, 5 μm. A representative image of four independent experiments is shown.

Small molecules targeting G-quadruplex can exert various effects on cells, including telomere shortening and uncapping, DNA damage and change in gene expression patterns^16^. The initial rationale for developing complex **1** was to stabilise telomeric G-quadruplexes and inhibit telomeric extension at the chromosomal ends as an anticancer strategy^17^. To this end, we measured telomeric extension using the Telomeric Repeat Amplification Protocol (TRAP) assay^18^. In addition to the conventional TRAP, a modified version, the SyBr Green-based RTQ–TRAP assay^19^, was performed to assess telomeric extension of MOLT-4 cells after the copper complex treatment. The ^tel^IC_50_ values (concentrations at which the telomeric extension activity was inhibited by 50%) determined by both conventional TRAP and RTQ-TRAP were >40 μM (Fig. 2A,B), which is more than 10Χ the IC_50_ against MOLT-4 cells determined by the MTT (24 h) cytotoxicity assay^13^. To fully understand the effect of complex **1** on telomeres, we measured the length of telomere of MOLT-4, human breast adenocarcinoma cells (MCF-7) and normal human fibroblast cells (IMR-90) at 1 and 7 days post-treatment with various concentrations of complex **1**. Our results showed no drastic shortening of telomeric length was observed even after 7 days of treatment (SI Figure S1). Next, we investigated if the G-quadruplex targeting complex **1** caused DNA damage using the DNA damage response marker γ-H2AX^20^ along with a telomere specific marker. Our results showed numerous hotspots dispersed over genomic DNA with some regions adjacent to telomeres (Fig. 2C). Altogether, we showed that complex **1** targets both telomeric DNA as well as the genomic DNA resulting in the nucleolytic DNA damage.

### Differential binding of complex 1 to various G-quadruplexes

G-quadruplex sequences occur on average once per 10 kb of the human genome. However, they have a higher incidence in promoters of oncogenes and telomeres^21^,^22^. Thus, many studies exploited these G-quadruplexes as rational targets for anticancer therapy^23^,^24^,^25^,^26^. Since complex **1** has been shown to bind selectively to the telomeric quadruplex, we decided to investigate its binding affinity to G-quadruplex forming sequences in selected well-established oncogene promoters and cancer-related genes using Fluorescence Intercalator Displacement (FID) titrations. With increasing amount of complex **1** added to a fixed amount of DNA, an increasing amount of DNA-bound thiazole orange (TO) was displaced. The binding affinity was measured by the concentration of the copper complex required to displace 50% of the TO (DC_50_). Our results revealed differential binding affinities of complex **1** to the G-quadruplex sequences in oncogene promoters. Complex **1** interacted with G-quadruplexes in *KRAS*, *c-Kit* and *c-Myc* promoters more strongly compared to double-stranded DNA and quadruplexes in *HIF1α*, *hTERT*, *VEGF*. (order of affinities: *KRAS*m(1:5:1) ~ *KRAS*m(1:9:1) > *c-Kit *> *c-Myc *> ds26 ~ *HIF1*α ~ *HTERT *~ *VEGF*) (Table 1). Overall, complex **1** has low affinity (DC_50 _> 2.5 μM) for the sequences investigated except for *KRAS*m(1:5:1) and *KRAS*m(1:9:1).

### *In situ* chemical affinity capture of *KRAS* and *c-Myc* promoter G-quadruplexes by complex 1

**Figure 3:**
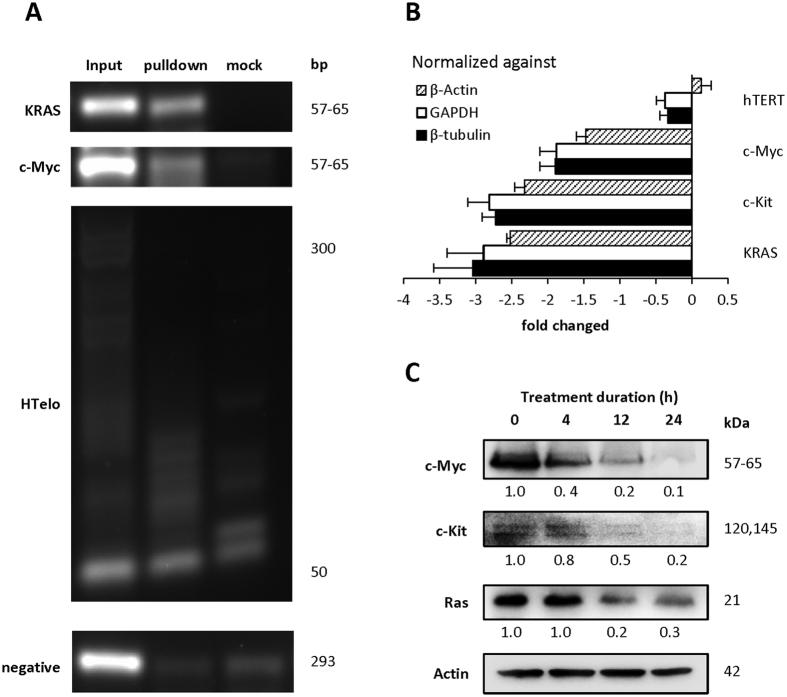
(**A**) Chemical affinity capture assay using complex 1* against G-quadruplex in MOLT-4 cells. Pulldown DNA samples were PCR amplified to investigate the interaction of complex 1* with *KRAS* and *c-Myc* promoters and HTelo. Input was the sonicated DNA fragments after 20 x dilution with ChIP dilution buffer. A representative image of five independent experiments is shown. (**B**) mRNA levels of *c-Myc*, *c-Kit* and *KRAS* normalized against *β-actin*, *GAPDH* and *β-tubulin* determined by realtime qPCR method. Quantification data are represented as mean ± SD of n = 5. (**C**) Protein levels of c-Myc, c-Kit and Ras determined using immunoblotting. The initial c-Kit protein level observed was very low even after enriched by membrane protein extraction, and decreased to an almost undetectable level after 24 h. Actin was used as a loading control. The number shown under each protein band indicates the average protein level from four independent experiments. A representative image of four independent experiments is shown.

To complement our *in vitro* observations, we performed a chemical affinity capture assay that couples ligand-click chemical capture and chromatin precipitation to identify the *in vivo* sites bound by small chemical molecules. To this end, we synthesized a derivative of complex **1** (complex 1*) that contains a 4-pentynyl group on the β position of the pyridine ligand (SI methods) in order to perform *in situ* Click chemistry^27^,^28^. To prevent potential DNA adducts after long period of interaction, MOLT-4 cells were sonicated after 2 h of treatment with 30 μM of complex 1* to generate short fragments of <1000 bp genomic DNA and Click reaction was performed in the presence or absence of the azide-biotin counterpart. After affinity pulldown using streptavidin beads, the DNA sequences bound onto the beads were amplified by PCR using specific primers for *KRAS*^23^ and *c-Myc*^29^ promoters and telomere^19^,^30^. PCR amplification of *KRAS* and *c-Myc* promoter showed significant enrichment in the azide-biotin treated samples compared to mock (without azide-biotin) samples (Fig. 3A; input represents sonicated DNA fragments used as positive control; a genomic locus from human chromosome 3 is used as negative control^31^). The observations showed that complex **1** was able to interact with accessible *KRAS* and *c-Myc* promoters *in vivo*, even though the FID data indicated that complex **1** does not bind *KRAS* and *c-Myc* G-quadruplexes strongly. On the other hand, there was no dramatic enrichment in the telomeric sequence in the pulldown samples, in contrast to the FID result.

### Complex 1 suppresses the expression of *c-Myc, c-Kit* and *RAS,* not *hTERT*

The effect of complex **1** on the expression of *c-Myc*, *c-Kit*, *KRAS and hTERT* were analysed by semi-quantitative real time PCR. MOLT-4 cells were first incubated with 3.0 μM (~MTT IC_50 (24_ _h)_) of the copper complex for 24 h before analysis by qPCR Our results showed differential mRNA expression levels of *c-Myc, c-Kit* and *KRAS* after treatment with complex **1**, which correlated with the FID findings, while there was no change in the expression of hTERT (Fig. 3B). The expression of *c-Myc* was reduced by twofold while a threefold reduction of gene expression was observed in both *c-Kit* and *KRAS.* Consistent with qPCR result, immunoblotting analysis showed reduced protein expression of c-Myc, c-Kit and Ras (Fig. 3C). Over 24 h of treatment, c-Myc protein dropped tremendously to an almost undetectable level, while c-Kit proteins were progressively reduced about five-fold, while Ras protein level remained high 4 h after treatment and reduced fivefold after 12 h treatment.

### Downregulation of Ras has strong inhibitory effect on both MEK/ERK and PI3K/Akt pathways

**Figure 4:**
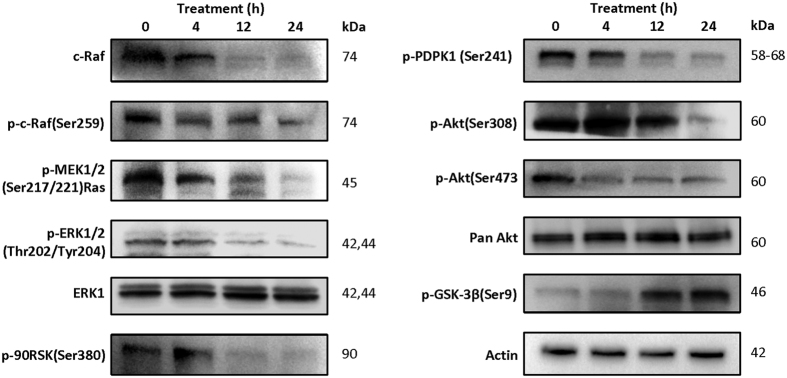
Downregulation of ERK and Akt signaling pathways by complex 1. Western blot analyses of ERK proliferation pathway (left panel) and Akt antiapoptotic pathway (right panel) in MOLT-4 cells over a 24-h period. Actin was used as a loading control. A representative image of three independent experiments is shown.

The mitogen-activated protein kinase (MAPK) pathway encompasses different signalling cascades of which the Ras-Raf-MEK-extracellular signal-regulated kinase 1 and 2 (ERK1/2) cascade is one of the most dysregulated in human cancers^32^. To delineate the signalling cascade by which complex **1** mediated its action, we examined downstream mediators of *KRAS* by Western blotting. Interestingly, complex **1** exerted a strong inhibitory effect on the MEK/ERK downstream anti-apoptotic pathway. c-Raf, which is a direct target of Ras, was significantly downregulated after treatment with complex **1** (Fig. 4, left panel). Raf, either through downstream MEK and ERK or independently of MEK and ERK, can induce the phosphorylation of proteins which control caspase-dependent apoptosis^33^. We also found the reduced phospho-activation downstream target MEK1/2, which stimulates ERK1/2. Activated phospho-ERK1/2 were strongly suppressed from 12 h of treatment with complex **1**. Further incubation of cells with complex **1** led to almost the complete abrogation of the phosphoERK1/2, whereas the total expression of ERK1/2 was not affected (Fig. 4, left panel). The phospho-activated ERK1/2 can modulate the activities of many kinases and transcription factors, such as 90 kDa ribosomal S6 kinase (90RSK) and c-Myc^34^. c-Myc is a critical transcription factor in malignant transformation by controlling genes involving in cellular proliferation, inhibition of differentiation and apoptosis, leading to its association with large number of cancer malignancies, and therefore have been heavily researched as a possible target for anticancer therapy^35^. Immunoblot analyses also revealed diminished active form of 90RSK (Fig. 4, left panel) as well as c-Myc protein (Fig. 3C panel) after 12 h of the copper complex treatment.

Ras is also able to induce the membrane translocation and activation of the p110 subunit of PI3K. PI3K converts phosphatidylinositol-4,5-phosphate (PIP2) into phosphatidylinositol-3,4,5-phosphate (PIP3) which results in the membrane localization of 3-phosphoinositide-dependent protein kinase-1 (PDPK1) via its pleckstrin homology (PH) domain^36^. Akt, also known as protein kinase Bα, is subsequently recruited to the plasma membrane by its PH domain and is phosphorylated at residues by T308 and S473 by PDPK1. This PI3K/Akt pathway is one of the most frequently activated signalling pathways in many cancers, and is responsible for the survival, metastasis and therapeutic resistance of cancers^37^. The PI3K/Akt pathway is also activated by the c-Kit receptor tyrosine kinase^38^, which we had observed to have reduced protein level through treatment with complex **1** (see above and Fig. 3C). Hence, we performed similar immunoblot analyses on the Akt antiapoptotic pathway. Results revealed that the downstream mediators of the PI3K cascade such as 3-phosphoinositide-dependent protein kinase-1 (PDPK1), Akt, and glycogen synthase kinase 3β (GSK-3β) were altered (Fig. 4, right panel). We also found that complex **1** treatment did not affect the expression level of total Akt, but significantly down-regulated the phosphorylated Akt. Reduced activation of Akt signalling results in the activation of proapoptotic factors that induce the expression of proapoptotic factors such as Fas ligand through its caspase-8 extrinsic pathway^38^. GSK3 inhibition (phosphorylated form) by Akt also prevents the translocation of the cytoplasmic signalling molecule β-catenin to the nucleus, which impedes the expression of Cyclin D1 and hence cell cycle progression^39^. Hence, complex **1** treatment reduced the activation of the pro-survival Akt pathway, which would have a direct impact on the survival of the cancer cells.

### Complex 1 triggers apoptosis in MOLT-4 cells

**Figure 5:**
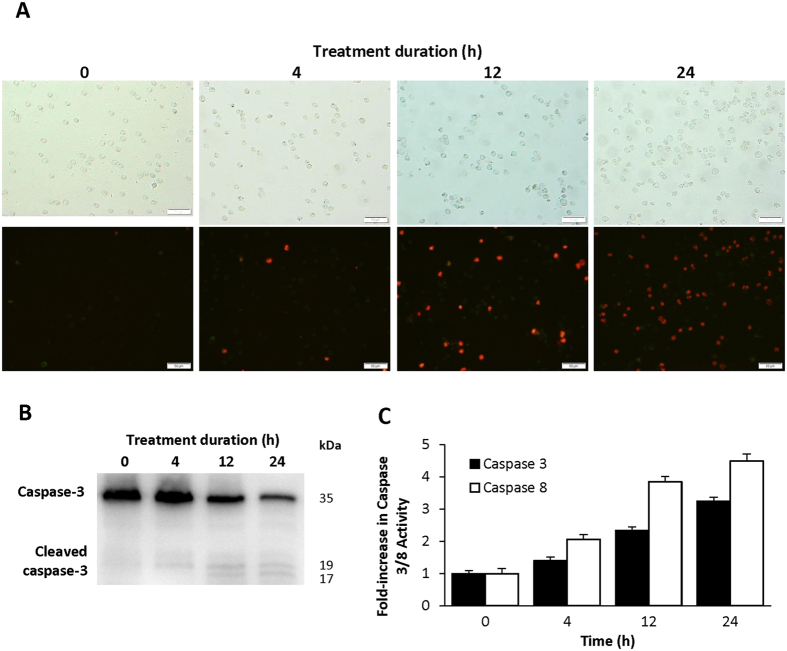
Assessment of apoptosis in MOLT-4 cells treated with 3.0 μM complex 1 over 24 h. (**A**) Treated cells visualized under bright field (upper panel) and annexin-V-FITC and propidium iodide staining (lower panel). Scale bars 50 μm. A representative image of four independent experiments is shown. (**B**) Activation of caspase 3 over 24 h after treatment. A representative image of three independent experiments is shown. (**C**) Caspase 3 and 8 activities, determined by the measurement of the colorimetric substrates Ac-DEVD-pNA and Ac-IETD-pNA in 96-well microplates incubated at 37 °C for 1 h. The increase in caspase activity is expressed in fold change over the treatment period. Quantification data are represented as mean ± SD of n = 6.

The failure to activate the apoptotic programme plays an important part in tumour cells^40^. Enhanced survival signals induced by several receptors, such as c-Kit receptor tyrosine kinase, are mediated mainly through the Akt and ERK pathways^41^. We hypothesized that the reduced activity of these pathways in complex **1** treated MOLT-4 cells stimulated increased susceptibility to antitumor drugs and consequently apoptosis. To confirm this hypothesis, we examined the apoptosis of MOLT-4 cells by annexin-V-FITC and propidium iodide (PI) staining over 24 h. Upon treatment with complex **1**, annexin-positive apoptotic cells were detected as early as 4 h, and more cells progressed into late apoptosis after 12 h (Fig. 5A). Western blot analysis also confirmed a time dependent decrease in the 35 kDa caspase-3 level and the gradual appearance of the cleaved fragments, indicating caspase-dependent apoptosis of MOLT-4 cells treated with the complex **1** (Fig. 5B). The caspase 3 activity was also measured using a colorimetric assay, which showed an increase of caspase activity at 12 and 24 h post-treatment (Fig. 5C). Fluorescence cell imaging on annexin-positive cells showed time dependent trigger of apoptosis in correspondence with the appearance of cleaved caspase-3. The deregulation of the PI3K/Akt pathway may lead to the extrinsic apoptotic pathway which relies on the activation of the caspase-8 initiator^41^. Thus, we analysed the activation of caspase-8. Indeed, a more prominent activation of caspase-8, than caspase 3, was detected (Fig. 5C).

### Complex 1 enhances drug sensitivity of MOLT-4 cells

**Figure 6:**
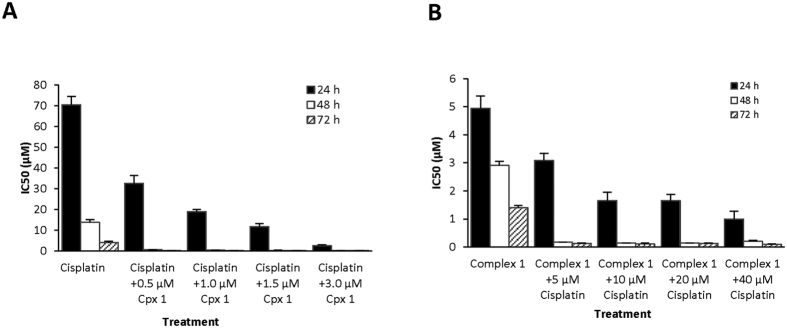
Cytotoxicity of combinations of complex 1 and cisplatin against MOLT-4 cells. (**A**) IC_50_ of cisplatin with various sub-lethal concentrations of complex **1 (**Cpx **1)** over 24, 48, 72 h treatment. Quantification data are represented as mean ± SD of n = 6. (**B**) IC_50_ of complex **1** with different sub-lethal concentrations of cisplatin over three-day treatment. Quantification data are represented as mean ± SD of n = 6.

The apoptotic effect of cisplatin is mediated through its DNA-adduct formation, which halts cellular processes such as replication and transcription, leading to prolonged G2 phase cell cycle arrest through deregulating the p53 tumour suppressor^42^. We showed above that complex **1** targets other key oncogenes and their downstream signalling pathways. Thus, it is conceivable that complex **1** can augment the anticancer activity of cisplatin, allowing a lower dose of cisplatin to eliminate cancer cells effectively without intolerable side-effects. Thus, we investigated the cytotoxic effects of cisplatin in combination with fixed sub-lethal concentrations of complex **1** on MOLT-4 cells. For cisplatin treatment alone, a high IC_50 (24 h)_ value of 70.4 μM was obtained after 24 h exposure. The IC_50 (48 h)_ decreased tremendously over the next 2 days, suggesting a relatively slow apoptotic action of cisplatin through its DNA-damaging effect. With the addition of complex **1**, the IC_50 (24 h)_ of cisplatin was reduced in a dose-dependent manner to 2.55–32.5 μM (Fig. 6A). The effect was more pronounced when MOLT-4 cells were incubated with both compounds for 48 h and 72 h, with almost 100% inhibition at 1.0 μM of cisplatin and 0.5 μM of complex **1**. Similarly, the effect of complex **1** treatment together with fixed sub-lethal concentrations of cisplatin was evaluated. The IC_50_ of the copper complex also decreased with the addition of cisplatin in a dose-dependent manner within the first 24 h, and the impact was more evident at 48 h and 72 h, where almost all cells were killed at 0.2 μM of complex **1** and 5 μM of cisplatin (Fig. 6B).

## Discussion

**Figure 7:**
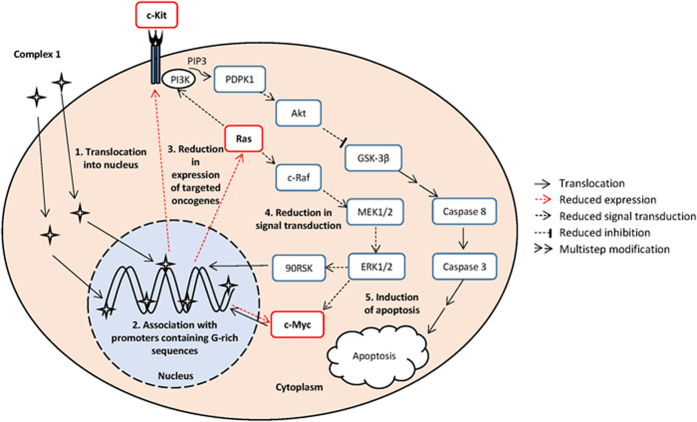
Schematic diagram of the cellular and molecular mechanisms of complex 1 induced apoptosis in MOLT-4 cells. The sequential events are (1) translocation of complex **1** into the nucleus, (2) association with promoters containing G-rich sequences, (3) reduction in gene expression of the targeted oncogenes, such as *c-Kit*, *Ras*, and *c-Myc*, resulting in reduced protein levels, (4) reduction in c-Kit and Ras-mediated MEK-ERK and PI3K-Akt signal transduction, (5) activation of caspase-dependent apoptosis.

Our observations suggest that complex **1** enters the cells and translocates into the nuclei, where it may interact with accessible G-quadruplex DNA on promoters of key oncogenes. The complex causes DNA damage at multiple genomic loci and downregulates the transcription of several genes that contain G-quadruplexes. The above effects lead to the reduced activity of anti-apoptotic PIK3/Akt and pro-mitogenic MAPK cascades (Fig. 7).

Although the FID data indicated that complex **1** binds with good affinity to HTelo, the *in vivo* data showed that the complex does not have a significant effect on telomeres, i.e. it interacts weakly with HTelo G-quadruplexes in cells. The reasons for this discrepancy could include differences in telomeric DNA topology and the inaccessibility of chromatin to complex **1** in cells, where telomeres are attached to the nuclear matrix and interact with various telomere-binding proteins.

The KRAS G-quadruplexes used in the FID studies contained two one-base external loops and a nine-base [KRAS m(1:9:1)] or five-base [KRASm(1:5:1)] external loop^23^. These sequences closely resemble those found in the *c-Myc* G-quadruplex, and adopt the same parallel topology similar to the *c-Myc*^43^*, c-Kit*^44^*, VEGF*^45^*, TERT*^46^ and *HIF1α*^47^ promoter quadruplexes. The binding affinities of complex **1** to these quadruplexes vary greatly, however, suggesting that other parameters may contribute to its affinity to G-quadruplexes. Indeed, differential binding affinities of similar small molecules to different quadruplex topologies have also been reported^48^. It is tempting to speculate that the length, sequence and flexibility of the external loop strongly influence the binding affinity and selectivity. We are currently investigating the correlation between binding affinity of small molecule metal complexes and the length and sequence of the external loops of the G-quadruplexes, which could facilitate the design of small molecules that target specific G-quadruplexes.

Nearly one third of tumour types have undergone an activating mutation in *RAS* genes that lead to the expression of constitutively active Ras proteins^49^. Moreover, the c-Myc transcription factor has a pivotal function in growth control, differentiation and apoptosis and is overexpressed in 80% of solid tumors^50^. This transcription factor orchestrates the expression of 10–15% of all cellular genes. Consequently, the constitutive expression of the *Ras* and *c-Myc* in human cancers is frequently associated with tumour aggression and poor clinical outcome^51^. The ability of complex **1** to suppress the expression of *Ras* and *c-Myc* is thus a significant finding. Indeed, we have shown that the complex can effectively induce apoptosis through the caspase-dependent pathway by reducing the expression of *Ras* and *c-Myc*, and by the inhibition of Ras-ERK and PI3K-Akt pathways. In view of the large numbers of genetic mutations and heterogeneity in many human cancers, therapies involving such targeting of multiple pathways may be more efficacious than single-target therapies.

An advantageous collateral effect of complex **1** is its ability to sensitize cancer cells to cisplatin, which would lower the effective antitumor concentration, and positions complex **1** as a potential adjunctive treatment. Whether other copper-based small molecules exhibit this role remains unclear and clearly warrants further investigation. With the emergence of chemoresistant cancer stem cells, and given the genetic heterogeneity of primary and recurrent cancers, multi-modal therapy has gained tremendous traction and is likely to be more useful than traditional single-drug approach^52^.

In conclusion, our study highlights the potential of small molecule copper complexes to cause catastrophic DNA damage and differentially regulate oncogenic pathways. We also envisage that using Chem-Seq, a method that employs chemical affinity capture coupled with massively parallel DNA sequencing, will reveal further insights into the genome-wide interaction of small molecules that is necessary for a full appreciation of the potential of copper-based complexes.

## Methods

### Cell Culture

The acute lymphoblastic leukaemia MOLT-4 (CRL-1582) suspension cell line was obtained from ATCC and maintained in RPMI-1640 medium (PAN Biotech) supplemented with 10% (v/v) heat-inactivated foetal bovine serum (Gibco) and antibiotics (100 U/ml penicillin and 100 U/ml streptomycin) (PAN Biotech), at 37 °C in a humidified atmosphere of 5% CO_2_.

### Cellular Uptake Protocol

Complex **1** was first prepared as previously reported^13^. 5 × 10^6^ MOLT-4 cells seeded in 25 cm^2^ culture flasks for 1 h were treated with complex **1** (3.0 μM) at 37 °C for 24 h. At various time points, the cells were collected and washed with phosphate buffered saline (PBS) twice before splitting the cell suspension into four parts. One part was used to analyse the metal content in the whole cell and the second part was subjected to subcellular fractionation using NE-PER Nuclear and cytoplasmic extraction reagents (Life Technologies). The left over pellet after extracting the nuclear fraction is referred to as the insoluble residue. The third part was used to isolate intact nuclei according to an established protocol^53^. The last portion of the cells was used to perform cell count and whole cell protein quantification using Bradford Assay (Bio-Rad). Various cellular and subcellular components were mineralized with 0.5 ml 65% HNO_3_ at 95 °C and the final volume was adjusted to 2 ml using double distilled water. Copper concentrations were determined by graphite furnace atomic absorption (GFAA) spectrometry using a Perkin Elmer PinAAcle 900T spectrometer. After background correction, copper levels were expressed as ng copper/ mg of cellular protein. Results are presented as the mean of three determinations for each data point with five separate experiments.

### Telomeric Repeat Amplification Protocol (TRAP) assay

The TRAP assay was performed using the TRAPeze Telomerase Detection Kit (Chemicon) on lysates of MOLT-4 cells. The lysates were treated with different concentrations of complex **1** in the presence of TS [5′-AATCCGTCGAGCAGAGTT-3′] and ACX [5′-GCGCGG(CTTACC)3CTAACC-3′] primers, followed by PCR amplification of the extended telomeric repeats, resolution of the amplified bands by gel electrophoresis and visualization using a fluorescent dye^18^. In addition to the traditional protocol, telomerase activity was also measured by SyBr Green-based realtime quantitative PCR (RTQ-TRAP)^19^. Briefly, 2 × 10^6^ cells was lysed at 4 °C for 30 min in 50 μl of CHAPS buffer containing RNase inhibitor. The lysate was then centrifuged at 12 000 × *g* for 30 min at 4 °C, and the supernatant was collected. The protein concentration was measured using the Bradford Assay (Bio-Rad). Each TRAP reaction mixture (20 μl) contained 1x SYBR Green buffer (Kapa Biosystems), 0.15 μg of TS, 0.1 μg of ACX, an additional 100 mM of KCl, and complex **1** at various concentrations. The reaction mixture was first incubated at 30 °C for 30 min to allow the telomerase in the protein extracts to elongate the TS primer by adding TTAGGG repeat sequences. The RT-PCR was then started at 95 °C for 10 min (to activate the hot-start DNA polymerase as well as to deactivate complex **1** to prevent it from inhibiting PCR process), followed by a 40-cycle amplification (94 °C for 30 s, 59 °C for 30 s, and 72 °C for 60 s). The fluorescence threshold was calculated as 10 SD of the baseline fluorescence intensity at the default setting of 3–15 cycles. Telomerase activity was calculated based on the threshold cycle (Ct), where 100% was taken for the Ct with control vehicle treatment (0 μM), and 0% when the fluorescence threshold did not reach the fluorescence threshold. ^tel^IC50 is at the concentration when telomerase activity is at 50% (ΔCt = 1) compared to the control. Results are presented as the mean of three replicates for each data point with four separate experiments, and heat inactivated protein extracts were used as negative controls.

### G-quadruplex Fluorescence Intercalator Displacement (FID) Assay

FID titrations were performed at room temperature using a fluorescence spectrophotometer (LS50B, Perkin Elmer)^54^. Briefly, G-quadruplex oligonucleotides of human telomere (HTelo), selected oncogene promoters (c-Myc, c-Kit, KRAS m(1:9:1), KRAS m(1:5:1), VEGF) and cancer related genes (HIF1α and hTERT) (Supplementary Table S1) were first annealed at 20 μM by heating to 90 °C for 5 min and cooling to room temperature overnight in 60 mM potassium buffer containing 50 mM potassium chloride and 10 mM potassium cacodylate (pH 7.4). This was further diluted with the potassium buffer to the final concentration of 0.25 μM before adding thiazole orange (TO) (stock solution of 500 μM in DMSO) to its final concentration of 0.625 μM. After mixing, stock solution of 500 μM complex **1** in DMSO was titrated stepwise to obtain 0–10 equivalence of the DNA concentration. For each addition, an equilibration time of 5 min was given before the emission spectrum was recorded between 510 and 750 nm with an excitation wavelength of 501 nm. The fluorescence intensity (peak area) was integrated and corrected for the background contribution by subtraction. The intensity data were plotted using the Stern–Volmer equation^13^ to determine the DC_50_ values (concentration of test compound causing 50% TO displacement). The average DC_50_ values were obtained from four sets of independent experiments.

### Genomic DNA extraction and Southern blotting to measure telomere length

Genomic DNA was extracted using Gentra Puregene Genomic DNA Purification Kit (Qiagen). For telomere length measurement, the genomic DNA was digested with HphI and MnlI at 37 °C for 16 hr. The DNA blot was hybridized with ^32^P-labeled (TTAGGG)_6_ oligonucleotides as previously described^55^.

### Immunofluorescence staining

After treatment with 3 μM of complex **1** for 24 h, MOLT-4 cells were fixed with 4% paraformaldehyde in 1x PBS for 10 min before permeabilization with KCM Buffer (120 mM KCl, 20 mM NaCl, 10 mM Tris pH 7.2, 0.5 mM EDTA, 0.1% (v/v) Triton X-100) for 10 min at room temperature. After blocking (150 mM NaCl, 20 mM Tris pH7.2, 2% BSA, 0.2% (v/v) Normal Goat Serum, 0.1% (v/v) Triton-X 100) for 30 min, the cells were incubated with mouse anti-γH2AX (1:1000 dilution, Millipore, 05-636) blocking buffer for 1 h. Subsequently the cells were washed thrice with KCM buffer and incubated with Alexa Fluor_568_-conjugated goat anti-mouse (1:1000, Invitrogen, A11031) for 40 min. To stain for telomere, the cells were subjected to dehydration in ethanol series (3 min 70% >2 min 90% >2 min 100%), incubating with 10 nM TelC-FITC in 70% deionized formamide, 10 mM Tris pH 7.5, 4 mM Na_2_HPO_4_, 0.5 mM Citric Acid, 1.25 mM MgCl_2_, 1% BSA at 80 °C for 3 min. before leaving the cells to hybridize at room temperature overnight. After washing thrice with 70% formamide, 10 mM Tris pH 7.5, the cells were rinsed thrice with Tris-buffer saline pH 7.5 with 0.08% Tween-20, before Tyramide Signal Amplification was carried out using TSA^TM^ Kit (Life Technologies, T20912) according to manufacturer’s protocol. Nuclei were visualized by DAPI staining (Sigma-Aldrich). Images were acquired on a Zeiss LSM 7 ELYRA PS.1 system (Carl Zeiss, Pte. Ltd., Singapore).

### *In situ* small molecule chemical affinity capture

A 4-pentnyl derivative of complex **1** (complex 1*) was first synthesized (SI Methods). 2 × 10^6^ MOLT-4 cells were incubated with 30 μM of complex 1* for 2 h and then treated with 1% paraformaldehyde in phosphate buffered saline for 10 min before being pelleted (4 min at 4000 rpm) and washed thrice with ChIP dilution buffer (1.1% Trixon-X 100, 1.2 mM EDTA, 16.7 mM Tris, pH 8.1, 167 mM NaCl). The cells were then sonicated in lysis buffer (10 mM EDTA, 50 mM Tris, pH 8.1, 1% SDS) at 2 × 10^6^ cells /mL to generate 300–1000 bp DNA fragments (used as input after 20x dilution). 50 μL of the resultant DNA fragments were subjected to Click-iT^®^ (Invitrogen, C10276) in the presence or absence (mock) of 2 μL of 4 mM Biotin-azide (Invitrogen, B10184) according to the manufacturer’s protocol. After diluting the final mixture with 1 mL of ChIP dilution buffer, 100 μL of streptavidin agarose resin (Thermo Scientific, 20357) was added and the mixture was incubated overnight at 4 °C. After washing thrice, 100 μL of ChIP dilution buffer was added and the mixture was incubated at 65 °C for 4 h before validating the presence of specific DNA sequences by PCR amplification using specific primers. The PCR amplification was started at 94 °C for 3 min, followed by a 40-cycle amplification (94 °C for 30 s, 55 °C (*KRAS*)/63.5 °C (*c-Myc*)/62 °C (HTelo)/53 °C (negative control) for 30 s, and 72 °C for 30 s). The amplified products were resolved in 2% (4.5% for HTelo) agarose gel in Tris-Borate buffer (pH 8.2) and visualized with SyBr Gold (Invitrogen, S11494) using a Chemi-doc MP system (Bio-Rad). Results presented are collected from at least four independent experiments.

### Realtime qPCR

Total RNA was first isolated using RNeasy mini kit (Qiagen) from MOLT-4 cells after 24 h treatment with 3.0 μM of complex **1**, followed by cDNA synthesis using 2 μg of total RNA by Qscript cDNA supermix (Quanta Biosciences). The resulting cDNA was amplified in SYBR green real-time quantitative PCR assays (Kapa Biosystems) with validated primers specific for each gene of interest, as shown in Supplementary Table S2. The PCR cycling was performed with mini-opticon (Bio-Rad) with a 40-cycle amplification followed by melting-curve analysis. The relative differences in RNA expression in samples with complex **1** and vehicle solvent treatment was evaluated by the comparative threshold cycle (Ct) method. Briefly, Ct values are first normalized to that of housekeeping genes (e.g. *β-tubulin*, *GAPDH* and *β-actin*) in the same sample (ΔCt), and then the differences in ΔCt values between each treatment and control group (ΔΔCt) were used to calculate the fold changes in expression (=2^ΔΔCt^) in each sample.

### Western blot analysis for protein expression and signalling pathways

Five million MOLT-4 cells were seeded in 25 cm^2^ culture flasks for 1 h before being treated with 3.0 μM of complex **1**. Treated cells were collected at indicated time points, washed twice with PBS, and then lysed in RIPA buffer on ice with protease inhibitors (Thermo Scientific) and phosphatase inhibitors (Sigma). After determining the protein concentration using the Bradford Assay (Bio-Rad), 30–50 μg of proteins were separated by 10–15% SDS–PAGE, transferred to PVDF membranes (Bio-Rad), and then probed with primary antibodies. Before and during the probing with antibodies, bovine serum albumin (BSA) instead of non-fat milk was used in Tris-buffer saline with 0.1% Tween20 (TBST) to avoid non-specific binding of the antibodies to phosphoproteins found in milk which would hinder signal detection. Primary antibodies against c-Myc (1:1500 dilution), c-Kit (1:1000 dilution), RAS (1:2000 dilution), Phospho-AKT pathway (1:2000 dilution), phospho-ERK1/2 pathway (1:2000 dilution) (Cell Signalling Technology) and caspase-3 (1:4000 dilution, Abcam) were used to evaluate the protein expression, signalling pathways and apoptosis, respectively. Actin (1:5000 dilution, Abcam) was used as a loading control. Horseradish peroxidase (HRP) – conjugated anti-rabbit IgG (Cell Signalling Technology) was used as the secondary antibodies. The respective protein levels were visualized with SignalFire ECL Reagent (Cell Signalling Technology) using a Chemi-doc MP system (Bio-Rad). Results presented are collected from at least three independent experiments.

### Assessment of apoptosis

One million MOLT-4 cells were first seeded in a 6-well sterile culture plate for 1 h before being treated with complex **1** (3.0 μM) or solvent control at 37 °C, 5% CO_2_ for 0, 4, 12, and 24 h. At indicated time points, cell death was determined by staining treated cells with annexin-V-FITC and counterstained with propidium iodide (PI) (Clontech). Annexin-V-FITC binds to the exposed phosphatidylserine (PS) on the outer plasma membrane of cells undergoing apoptosis, while PI is excluded by viable cells with intact membranes. Cells positive for annexin-V-FITC but negative for PI are undergoing early stages of apoptosis. Treated cells were washed twice with PBS and stained with annexin-V-FITC and PI. Cells were analysed by an Olympus BX51 microscope equipped with a DP70 camera under 20x magnification. Images showed are collected from three independent experiments.

In addition to annexin-V staining, caspase 3 and 8 activities were quantified under the same treatment regime using the same number of cells according to instructions of the colorimetric assay kits (BD Bioscience Clontech). Briefly, cell pellets collected from each well were lysed in 50 μl cell lysis buffer after treatment, centrifuged at 12 000 × *g*, and 50 μl of supernatant were mixed with 50 μl 2x reaction buffer and incubated with the caspase 3 or 8 substrate, Ac-DEVD-pNA (50 μM) or Ac-IETD-pNA (200 μM) in a 96-well microplate at 37 °C for 1 h. The caspase 3 or 8 activity were determined spectrophotometrically at 405 nm using a Benchmark Plus microplate spectrophotometer (Bio-Rad). Unit caspase activity was determined by the difference in optical density divided by the slope of standard curve, taking 0 h as 1x caspase activity. The average caspase activities are calculated using three sets of independent experiments.

### MTT Cytotoxicity Assay

MOLT-4 cells were plated in 96-well microplates at a density of 20,000 cells in 70 μl/well before various concentrations (0–125 μM) of complex **1** and/or cisplatin (Sigma) were added. The test compounds were prepared as 10 mM stock solutions in DMSO and diluted further to 1 mM using fresh culture medium. This was then serially diluted six times to give other working solutions in 10% DMSO (v/v) in culture medium. Ten microliters of each working solution was added to each test well. The final concentration of DMSO was 1.25% (v/v) in each well. After incubation for 24, 48 and 72 h, 20 μl/well of MTT (Sigma) solution (5 mg /ml) were added and the plates were incubated for 3 h before lysing solution (20% sodium dodecyl sulfate dissolved in 50% N,N-dimethylformamide, pH adjusted to 4.7 with acetic acid) was added to each well. After standing overnight, the absorbance of the solution in each well was read at 570 nm using a Benchmark Plus microplate spectrophotometer (Bio-Rad). The percentage inhibition of growth for each concentration of compound(s) was calculated from the absorbance and plotted against the concentration to give a graph from which the IC_50_ value (concentration of compound required to inhibit the growth of the cells by 50%) was determined. Each plate also contained a blank well (cell-free medium only), solvent control wells (cells and 1.25% (v/v) DMSO), drug colour control wells (test compound(s) and medium), and growth control wells (cells in medium only), which were used for background correction. Six replicate test wells were set up for each concentration of test compound(s).

## Additional Information

**How to cite this article**: Goh, Y.-Y. *et al.* Downregulation of oncogenic *RAS* and *c-Myc* expression in MOLT-4 leukaemia cells by a salicylaldehyde semicarbazone copper(II) complex. *Sci. Rep.* **6**, 36868; doi: 10.1038/srep36868 (2016).

**Publisher’s note:** Springer Nature remains neutral with regard to jurisdictional claims in published maps and institutional affiliations.

## Supplementary Material

Supplementary Information

## Figures and Tables

**Table 1:** Binding affinities and selectivity of complex 1 for various oligonucleotides, determined by the FID assay.

	DC_50_^a^ (μM)	Selectivity against ds26^b^
HTelo	1.01 (0.03)	9.81
KRAS m(1:5:1)	2.28 (0.10)	4.34
KRAS m(1:9:1)	2.35 (0.09)	4.22
c-Kit	3.58 (0.28)	2.77
c-Myc	7.15 (0.17)	1.39
ds26	9.91 (0.55)	—
HIF1α	10.20 (0.54)	0.97
hTERT	10.51 (0.61)	0.94
VEGF	11.58 (0.41)	0.86

^a^Standard deviation (n = 4) are shown in parentheses.

^b^Ratio of DC_50_ for oligonucleotides against ds26.
