# Supplementary material for: Downregulation of oncogenic RAS and c-Myc expression in MOLT-4 leukaemia cells by a salicylaldehyde semicarbazone copper(II) complex
Source: Sci Rep. 2016 Nov 14;6:36868. doi: 10.1038/srep36868 (PMC5107956; doi:10.1038/srep36868)
Supplement: Supplementary Information [file srep36868-s1.pdf]

Downregulation of oncogenic *RAS* and *c-Myc* expression in MOLT-4 leukaemia cells by a salicylaldehyde semicarbazone copper(II) complex

Yan-Yih Goh [1*], Yaw-Kai Yan [1], Nguan Soon Tan [2,3,4,5], Su-Ann Goh [6], Shang Li [6], You-Chuan Teoh [1], and Peter P.F. Lee [7]

[1]Natural Sciences & Science Education, National Institute of Education, Nanyang Technological University, 1 Nanyang Walk, Singapore 637616, Singapore, [2]School of Biological Sciences, Nanyang Technological University, 60 Nanyang Drive, Singapore 637551, [3]Lee Kong Chian School of Medicine, Nanyang Technological University, 50 Nanyang Avenue, Singapore 639798, Singapore, [4]Institute of Molecular and Cell Biology, 61 Biopolis Drive, Singapore 138673, Singapore, [5]KK Women's and Children's Hospital, 100 Bukit Timah Road, Singapore 229899, Singapore, [6]Duke-NUS Graduate Medical School, 8 College Road, Singapore 169857, Singapore, [7]Singapore Institute of Technology, 10 Dover Drive, Singapore 138683, Singapore

***Corresponding author**

E -mail: yanyih.goh@nie.edu.sg.

<u>**Supplementary Information**</u>

Methods

Synthesis of 4-pentynyl nicotinate. Nicotinic acid (0.948 g, 7.70 mmol) was added to a stirred solution of 4-pentyn-1-ol (0.647 g, 7.69 mmol) in freshly distilled dichloromethane (25 mL). *N, N'*-Dicyclohexylcarbodiimide (1.578 g, 7.65 mmol) and 4-(dimethylamino)pyridine (0.035 g, 0.29 mmol, 4 mol%) were then added to the mixture. The resultant suspension was stirred for 48 h in a stoppered round bottom flask at 25 °C. The reaction mixture (orange suspension) was then filtered and the yellow filtrate was evaporated under reduced pressure. The resultant residue was extracted using hexane (30 mL x 3) and the combined hexane extract was evaporated under reduced pressure. The white solid obtained was washed thoroughly with distilled water (30 mL x 3) and dried in a vacuum oven at 40°C for 4 h.

Yield: (314 mg, 1.66 mmol, 22%). Anal. Calcd for $C_{11}H_{11}NO_2$: C, 69.8; H, 5.8; N, 7.4. Found: C, 69.9; H, 6.0; N, 7.5. IR (KBr, cm^{-1}): ν(C=O) 1712 vs, ν(C≡C–H) 3253 vs, ν(C≡C) 2109 vw. ^{1}H NMR [$CDCl_3$, ppm]: 9.23 [1H, d, $^{4}J_{HH}$ = 2 Hz, py H_{α} (next to COO)], 8.78 (1H, dd, $^{3}J_{HH}$ = 5 Hz, $^{4}J_{HH}$ = 2 Hz, py H_{α}), 8.30 (1H, dt, $^{3}J_{HH}$ = 8 Hz, $^{4}J_{HH}$ = 2 Hz, py H_{γ}), 7.40 (1H, dd, $^{3}J_{HH}$ = 8 Hz, $^{4}J_{HH}$ = 5 Hz, py H_{β}), 4.48 (2H, t, $^{3}J_{HH}$ = 6 Hz, OCH_2), 2.40 (2H, td, $^{3}J_{HH}$ = 7 Hz, $^{4}J_{HH}$ = 3 Hz) $CH_2C{\equiv}C$, 2.03 (2H, quint, $^{3}J_{HH}$ = 7 Hz $CH_2\mathbf{CH_2}CH_2$), 2.01 (1H, t, $^{4}J_{HH}$ = 3 Hz C≡C–H).

Preparation of (2,4-dihydroxybenzaldehyde dibenzyl semicarbazone) (4-pentynyl nicotinate) copper(II) nitrate (Complex 1*). Chlorido(2,4-dihydroxybenzaldehyde dibenzyl semicarbazone)copper(II) (38.4 mg, 0.081 mmol) was dissolved in 10 mL of methanol in a round bottom flask. One molar equivalent of silver nitrate (13.8 mg, 0.081 mmol) was added, and the mixture (shielded from light) was stirred vigorously for 2.5 h before it was filtered through Celite. The filtrate (dark green solution) was evaporated under reduced pressure to approximately 3 mL. 1 molar equivalent of 4-pentynyl nicotinate (15.3 mg, 0.081 mmol) was dissolved in 1 mL of methanol and added to the dark green solution. The resulting solution was stirred at r.t. for 2 h. Cold diethyl ether (40-

50 mL) was added, and the mixture was stored overnight at 4°C. The precipitate formed was isolated by filtration, washed with cold diethyl ether, and dried under vacuum at 40°C for at least 4 h.

Yield: (35.3 mg, 0.051 mmol, 64 %). Anal. Calcd for $C_{33}H_{31}CuN_5O_8$. 1½ H_2O: C, 55.3; H, 4.75; N, 9.8. Found: C, 55.5; H, 4.9; N, 9.8. IR (KBr, cm^{-1}): ν(C=O) 1724 vs, ν(C≡C–H) 3279 vs, ν(C≡C) 2115 vw.

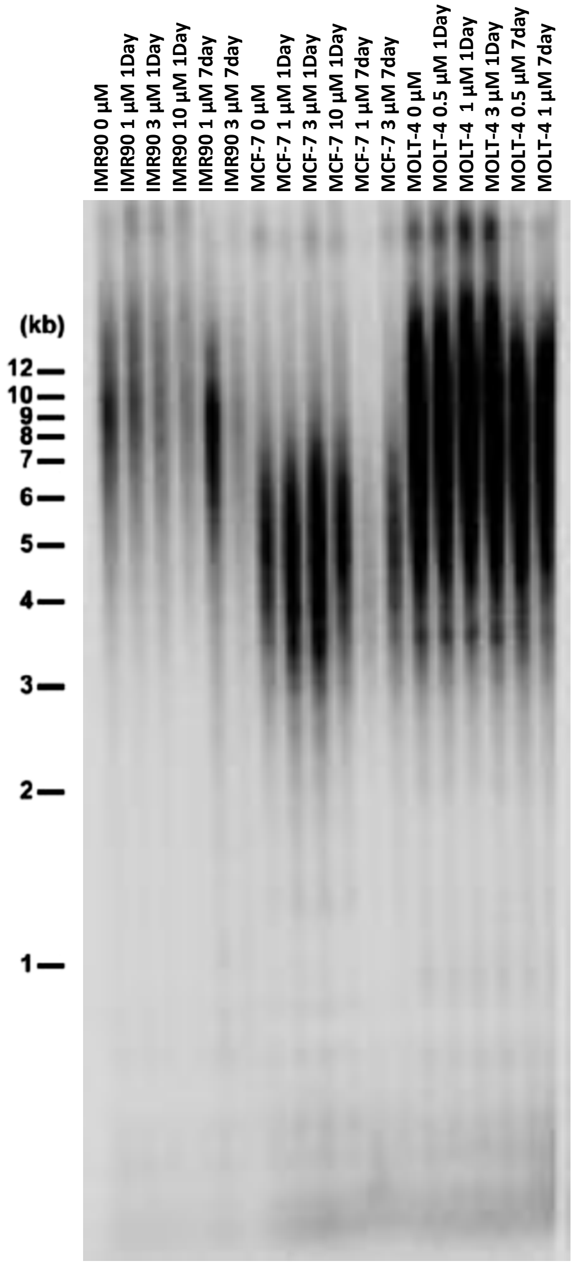

Figure S1. Short term treatment of complex **1** does not induced significant changes in telomere length. Normal human fibroblast cells (IMR90) and cancer cells (MCF-7 and MOLT-4) are treated with complex **1** at different dosages for the indicated time. The genomic DNA was purified and the telomere length was measured using Genomic Southern analysis probed with telomere specific probe.

Table S1. Nucleotide sequences used for Fluorescence Intercalator Displacement (FID) assay.

Name of sequence	Nucleotide sequence
HTelo	5'– AGGGTTAGGGTTAGGGTTAGGG –3'
KRAS m(1:5:1)	5'– GGGAGGGAAGGAGGGAGGG –3'
KRAS m(1:9:1)	5'– GGGAGGGAAGGAGGGAGGGAGGG –3'
c-Kit21	5'– CGGGCGGGCGCGAGGGAGGGG –3'
c-Myc	5'– GGGAGGGTGGGGAGGGTGGG –3'
ds26	5'– CAATCGGATCGAATTCGATCCGATTG –3'
HIF1α	5'– CGGGGAGGGGAGAGGGGGCGGGA –3'
hTERT	5'– GGGGAGGGGCTGGGAGGGCCC –3'
VEGF22	5'– CGGGGCGGGCCGGGGGCGGGGT –3'

Table S2. Nucleotide sequences of primer sets used for quantitative real time PCR and qualitative *in-situ* Chem-ChIP.

Gene	Primer sequences	Amplicon size (bp)	PrimerBank ID[1]
c-MYC	5'- GGCTCCTGGCAAAAGGTCA -3'<br>5'- CTGCGTAGTTGTGCTGATGT -3'	119	239582723c1
c-KIT	5'- CGTTCTGCTCCTACTGCTTCG -3'<br>5'- CCCACGCGGACTATTAAGTCT -3'	117	148005048c1
KRAS	5'- TGTGTCTCATATCAGGTTGACGA -3'<br>5'- CAAGAGTCGAGTGTGGTCTCA -3'	170	209529676c1
hTERT	5'- AAATGCGGCCCCTGTTTCT -3'<br>5'- CAGTGCGTCTTGAGGAGCA -3'	76	301129199c1
β-tubulin	5'- TGGACTCTGTTCGCTCAGGT -3'<br>5'- TGCCTCCTTCCGTACCACAT -3'	155	34222261c1
GAPDH	5'- GGAGCGAGATCCCTCCAAAAT -3'<br>5'- GGCTGTTGTCATACTTCTCATGG -3'	197	378404907c1
β-actin	5'- CATGTACGTTGCTATCCAGGC -3'<br>5'- CTCCTTAATGTCACGCACGAT -3'	250	4501885a1
KRASprom[2]	5'- TTCTCCCCGCCGGCGCTCGC -3'<br>5'- CTCGATTCTTCTTCAGACGG -3'	95	-
c-MYCprom[3]	5'- AGTGCTCGGCTGCCCGGCTGA -3'<br>5'- CTTTTCCCCCACGCCCTCTGC -3'	106	-
HTelo (TRAP assay)	5'- AATCCGTCGAGCAGAGTT -3'<br>5'- GCGCGGCTTACCCTTACCCTTACCCTAACC -3'	50-300	-
Negative (human chromosome 3[4])	5'- TAGGCTGGAGGTCGTGGTTA -3'<br>5'- CGGCGCTTTCGGATTAACT -3'	293	-

1 Wang, X., Spandidos, A., Wang, H. & Seed, B. PrimerBank: a PCR primer database for quantitative gene expression analysis, 2012 update. *Nucleic Acids Res* **40**, D1144-1149, doi:10.1093/nar/gkr1013 (2012).

2 Cogoi, S. & Xodo, L. E. G-quadruplex formation within the promoter of the KRAS proto-oncogene and its effect on transcription. *Nucleic Acids Res* **34**, 2536-2549, doi:10.1093/nar/gkl286 (2006).

3 Siebenlist, U., Hennighausen, L., Battey, J. & Leder, P. Chromatin structure and protein binding in the putative regulatory region of the c-myc gene in Burkitt lymphoma. *Cell* **37**, 381-391 (1984).

4 Thakur, R. K. *et al.* Metastases suppressor NM23-H2 interaction with G-quadruplex DNA within c-MYC promoter nuclease hypersensitive element induces c-MYC expression. *Nucleic Acids Res* **37**, 172-183, doi:10.1093/nar/gkn919 (2009).
